# Senloytics, dasatinib plus quercetin, reduce kidney inflammation, senescent cell abundance, and injury while restoring geroprotective factors in murine diabetic kidney disease

**DOI:** 10.1016/j.ebiom.2026.106124

**Published:** 2026-01-20

**Authors:** Xiaohui Bian, Zachary K. Snow, Caroline J. Zinn, Anastasia L. Bratulin, Khaled M. Elhusseiny, Tamar Tchkonia, James L. Kirkland, Yi Zhu, Sundeep Khosla, Seo Rin Kim, Lilach O. Lerman, LaTonya J. Hickson

**Affiliations:** a Division of Nephrology and Hypertension, Mayo Clinic, Jacksonville, FL, USA; b Department of Nephrology, Shengjing Hospital of China Medical University, Shenyang, China; c Center for Advanced Gerotherapeutics, Division of Endocrinology and Metabolism, Department of Medicine, Cedars Sinai Health Sciences University, Los Angeles, CA, USA; d Kogod Center on Aging and Division of Endocrinology, Mayo Clinic, Rochester, MN, USA; e Department of Cellular & Integrative Physiology, UT Health San Antonio, San Antonio, TX, USA; f Department of Physiology and Biomedical Engineering, Mayo Clinic, Rochester, MN, USA; g Division of Nephrology, Pusan National University School of Medicine, Yangsan, South Korea; h Division of Nephrology and Hypertension, Mayo Clinic, Rochester, MN, USA

**Keywords:** Fibrosis, Klotho, Diabetic nephropathy, Macrophage, Inflammation

## Abstract

**Background:**

Maladaptive inflammation and cellular senescence contribute to diabetic kidney disease (DKD) pathogenesis and represent important therapeutic targets. Senolytic agents selectively remove senescent cells and reduce inflammation-associated tissue damage. In our pilot clinical trial in patients with DKD, the senolytic combination dasatinib plus quercetin (D + Q) reduced systemic inflammation, senescent cell abundance, and macrophage infiltration in fat. However, D + Q senotherapeutic effects on diabetic kidney injury, senescence, inflammation, and geroprotective factors have not been established.

**Methods:**

Diabetes mellitus was induced with intraperitoneal streptozotocin in male C57BL/6J mice, followed by a 5-day oral gavage regimen of either D + Q (5 and 50 mg/kg, respectively) or vehicle. Kidney function and markers of injury, fibrosis, inflammation, cellular senescence, and geroprotective factors were measured. *In vitro* studies examined reparative effects of D + Q in high glucose-treated human renal tubular epithelial cells (HK2), endothelial cells (HUVECs), and U937-derived macrophages.

**Findings:**

D + Q improved kidney function and reduced markers of kidney injury (glomerular and tubular), fibrosis, senescence (p16^Ink4a^), macrophage- and senescence-associated inflammation (versus diabetic controls) without altering glucose levels. Additionally, geroprotective factors (α-Klotho, Sirtuin-1) increased. D + Q treatment *in vitro* reduced high glucose-induced senescence and inflammation (NF-κB) in HK2, HUVECs, and macrophages.

**Interpretation:**

A “hit-and-run” senolytic treatment with D + Q improved kidney function and mitigated murine DKD by modulating the inflammatory landscape, reducing senescent cell abundance, and restoring geroprotective factors. Taken together, the beneficial effects in kidneys of mice and prior systemic effects in humans, support the rationale for further clinical investigations applying D + Q to enhance healthspan in individuals with DKD.

**Funding:**

This research was supported by 10.13039/100000002National Institutes of Health (NIH): [DK123492, DK109134, and AG076537 (LJH); DK120292 and HL158691 (LOL); AG087387 (YZ); R37AG013925 (TT and JLK)]; NIDDK Diabetes Complications Consortium [DK115255, DK076169 (LJH)]; Mayo Clinic Robert D. and Patricia E. Kern Center for the Science of Health Care Delivery (LJH); TT and JLK are supported by a grant from the Hevolution Foundation (HF-GRO-23-1199148-3), Cedars-Sinai Medical Center, the Connor Fund, and Robert J. and Theresa W. Ryan.

**Research in context**

**Evidence before this study**

Cellular senescence is an increasingly recognised contributor to the pathogenesis of diabetes mellitus and its complications, including diabetic kidney disease (DKD), the leading cause of kidney failure worldwide. Senolytic agents, which selectively eliminate senescent cells, reduce inflammation and increase lifespan in non-DKD animal models. In a pilot clinical trial, the senolytic combination of dasatinib and quercetin (D + Q) cleared senescent cells in adipose tissue and reduced systemic inflammation in patients with DKD.

**Added value of this study**

Despite promising results of early phase clinical trials applying D + Q in humans with various conditions, their reparative effects in the diabetic kidney remain unclear. This study demonstrated in a mouse model of DKD that a single, oral 5-day regimen reduced kidney injury, senescence, and inflammation and improved kidney function compared to controls. Additionally, our *in vitro* experiments involving human kidney cells, endothelial cells, and macrophages confirmed that D + Q treatment blunted hyperglycaemia-induced senescence and suppressed nuclear factor-kB-associated inflammation.

**Implications of all the available evidence**

Clinical application has confirmed D + Q senolytic effects in human adipose tissue and circulating inflammation. Our preclinical study suggests promising reparative effects in the diabetic kidney and supports pursuit of additional clinical trials optimising D + Q regimens to therapeutically benefit individuals with DKD.

## Introduction

Diabetes mellitus, estimated to affect approximately 1.31 billion individuals worldwide by 2050,^1^ is the leading cause of kidney disease.^2^^,^^3^ A major complication of diabetes, diabetic kidney disease (DKD), results from multiple pathogenic processes including glucose toxicity, accumulation of advanced glycation end products (AGEs), oxidative stress (OS), renin-angiotensin-aldosterone system (RAAS) activation, and uraemic toxin exposure. These insults contribute to chronic sterile inflammation and the accumulation of senescent cells.^4^^,^^5^ Cellular senescence, an essentially irreversible state of proliferative arrest, is a response to a variety of stressors including metabolic stress, inflammation, oncogenic mutations, DNA damage, and mitochondrial dysfunction.^6^, ^7^, ^8^, ^9^ Stressors lead to induction of the cyclin-dependent kinase (CDK) inhibitors p16^INK4A^, p19^ARF^, and p21^CIP1^, and result in cell-cycle arrest at the G1/S cell-cycle checkpoint by enhancing checkpoint activity.^10^, ^11^, ^12^, ^13^, ^14^, ^15^ Despite cell cycle arrest, senescent cells remain metabolically active, are resistant to apoptosis, and develop a senescence-associated secretory phenotype (SASP) leading to organ dysfunction.^10^^,^^16^ The SASP is comprised of inflammatory cytokines, chemokines, growth factors, extracellular matrix-degrading proteins, and other factors that contribute to senescence-related inflammation, metabolic dysregulation, stem cell dysfunction, ageing phenotypes, and chronic diseases.^8^^,^^9^

In human DKD biopsy studies, senescent cell markers were detected in both early and advanced DKD stages and predominantly localised to tubular epithelial cells.^17^ Similarly, in streptozotocin (STZ)-induced DKD mouse models, podocyte^18^ and tubular senescent cells were observed in early and advanced stages of DKD.^18^, ^19^, ^20^ The presence of senescent cells early in DKD suggests that propagation of the SASP and senescence in neighbouring cells may drive disease progression rather than merely serving as bystanders. We previously reported that activin A, a key SASP component in senescent adipose tissue and a profibrotic marker, was highly expressed in the blood and urine of patients with DKD and inversely correlated with kidney function.^21^ In murine DKD we determined that antagonising activin a reduced senescent cell abundance and kidney injury.^22^ These findings implicate a pathologically conserved mechanism between mice and humans and suggest that targeting cellular senescence and the SASP may exert broad therapeutic effects in DKD.

Senolytics, agents that selectively clear senescent cells, are promising therapeutics for DKD.^6^^,^^23^ We previously demonstrated that the combination of dasatinib and quercetin (D + Q) has senolytic activity against a broad range of senescent cells, including adipocyte progenitors and umbilical vein endothelial cells, through effective targeting of senescent cell anti-apoptotic pathways (SCAPs).^24^^,^^25^ The senolytic and protective effects of D + Q were demonstrated in ageing-related animal disease models, including chronologically aged, radiation-exposed, progeroid Ercc^−1/Δ^, and diet-induced obese mice.^2^^,^^25^, ^26^, ^27^, ^28^, ^29^, ^30^, ^31^ Clinical translation has been positive, notably in early-stage trials in individuals with diabetes,^32^ idiopathic pulmonary fibrosis,^33^ Alzheimer's disease,^34^^,^^35^ and age-related bone loss.^36^ These investigations were extended to an early-phase pilot clinical trial in patients with DKD where we were among the first to demonstrate that D + Q decreased senescent cell burden and macrophage infiltration in adipose tissue and reduced circulating SASP factors.^32^ In Type-2 diabetic (db/db) mice D + Q promoted PPARα-mediated fatty acid oxidation leading to kidney repair.^37^ However, whether the senolytic therapy D + Q modulates cellular senescence, inflammation, and geroprotective factors (α-Klotho, Sirtuin-1) in the diabetic kidney is unknown. We therefore sought to characterise these effects in a murine model of DKD.

## Methods

### Animal experimental design

**Fig. 1:**
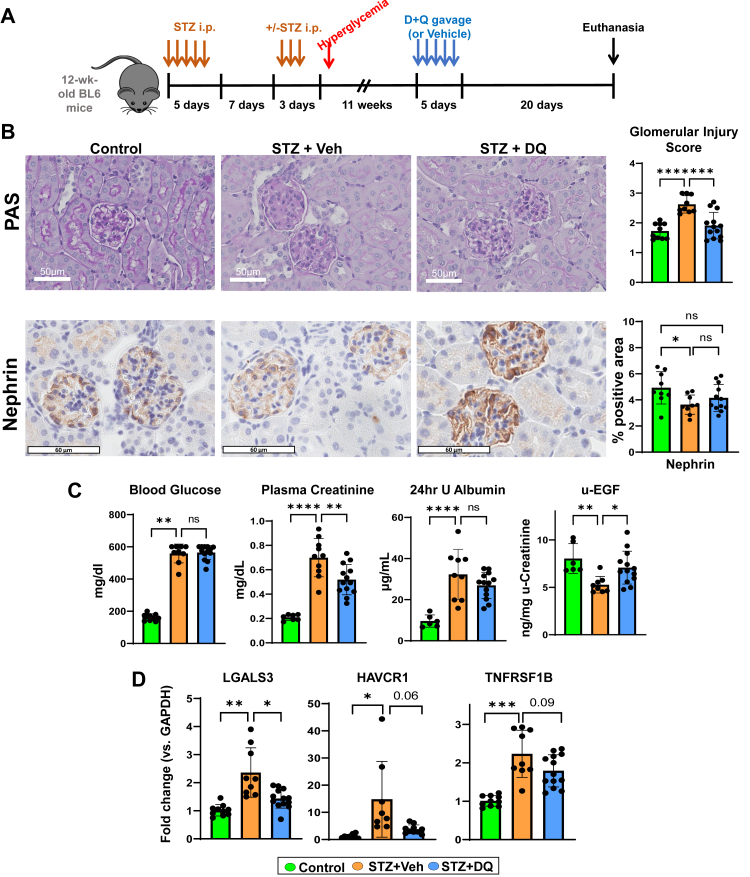
**D + Q improved kidney injury in STZ-induced diabetic mice. A)** Schematic of STZ-induced diabetic C57BL/6J mice treated with DQ for 5 days beginning 11 weeks after hyperglyceamia; euthanasia for tissue harvest at Week 29. **B)** Representative images and quantification of glomerular injury score in PAS and nephrin staining of paraffin-embedded kidney sections (5 μm) from Control, STZ + Veh, and STZ + DQ mice (Scale bars = 50 μm). **C)** Biochemical analyses and ELISA were performed to delineate effects of DQ on blood glucose, kidney function, albuminuria, and injury marker, u-EGF. **D)** qRT-PCR of kidney tissue for kidney injury biomarkers—*LGALS3*, *HAVCR1*, and *TNFRSF1B*. All data are shown as mean ± SEM. ∗p < 0.05, ∗∗p < 0.01, ∗∗∗p < 0.005, and ∗∗∗∗p < 0.001. Data were analysed with unpaired t-tests or ANOVA where appropriate. DQ: dasatinib plus quercetin, PAS: Periodic Acid-Schiff, u-EGF: urinary epidermal growth factor, LGALS3: galectin 3, HAVCR1: hepatitis A virus cellular receptor 1 (KIM-1), TNFRSF1B: TNF receptor superfamily member 1B (TNFR2).

All animal experiments were approved by the Mayo Clinic Institutional Animal Care and Use Committee. Twelve-week-old male C57BL/6J mice (Jackson Lab, Bar Harbour, ME, RRID:IMSR_JAX:000664) received intraperitoneal streptozotocin (Cayman Chemical Company CAT #13104) 50 mg/kg/day for 5 (+3) consecutive days (Fig. 1A).^38^ Female mice historically have been resistant to STZ-induced diabetes and consistent DKD injury, therefore they were not included in this study.^39^^,^^40^ Mice with fasting glucose >250 mg/dL were considered diabetic. At day 100, diabetic mice were randomised to a 5-day oral gavage regimen of either D + Q (5 and 50 mg/kg, respectively, n = 13) or vehicle (60% Phosal 50 PG, 30% PEG400, 10% Ethanol, STZ, n = 9). Doses of D and Q were based on previous studies.^2^^,^^25^^,^^37^^,^^41^, ^42^, ^43^ Controls were age-matched C57BL/6J mice treated with vehicle gavage daily for 5 days (n = 10). Urine was collected over 24 h in metabolic cages before euthanasia by terminal cardiac blood sampling under 2% isoflurane anaesthesia which took place 20 days after treatment. The kidneys were harvested, weighed, and cut into two halves to be frozen fresh or preserved in formalin. Sample sizes were selected based upon preliminary data and our prior investigations applying senolytics.^2^^,^^44^ Over the course of their lifespan, animals were monitored to reduce pain, suffering, and distress.

### Ethics

All animal experiments were approved by the Mayo Clinic Institutional Animal Care and Use Committee (A00004821).

### Biochemical analysis

Fasting blood glucose was measured with a glucometer (Accu-Chek Aviva) and urinary albumin by ELISA using Albuwell (Exocell, 1011, Philadelphia, PA). Kidney function was assessed with plasma creatinine using a commercial kit (Arbour Assay, KB02 DetectX® Serum Creatinine kits). Urine creatinine and epidermal growth factor (EGF) were measured with commercial kits (Arbour Assay, K002, DetectX® Urine Creatinine kits, MILLIPLEX® MKI2MAG-94K Mouse Kidney Injury-Toxicity Multiplex Assay, respectively). Sirtuin-1 protein was measured in homogenised kidney tissue using a commercial kit (Abcam, ab206983, Waltham, MA).

### Histology and immunohistochemistry

Kidney tissue was fixed with 10% formalin, embedded in paraffin, stained with periodic acid-schiff (PAS) or Trichrome reagents, and examined under an optical microscope. Glomerular injury score was calculated in PAS. Details for methods used to determine these scores are provided in Supplemental Methods. Expression of nephrin (1:500, ab216341, Abcam, RRID:AB_2864307), fibronectin (1:2500, ab268020, Abcam, RRID:AB_2941028), MCP-1 (1:500, ab25124, Abcam, RRID: AB_448636), p16 (1:100, ab189034, Abcam, RRID:AB_2737282), and α-Klotho (1:400, MA5-32784, Invitrogen, RRID:AB_2810060) were examined in paraffin-embedded 5 μm renal tissue sections by IHC analysis. In each section ≥10 images were captured randomly with blinding to groups (See Supplemental Methods).

### In vitro studies

To explore D + Q potential therapeutic mechanisms, injury models were established in kidney tubular cells, endothelial cells, and monocytes in a diabetic *milieu.* Cells included human proximal tubular epithelial cells (HK2; CRL-2190, American Type Culture Collection, RRID:CVCL_0302), human umbilical vein endothelial cells (HUVEC, 200K-05f, Cell Applications), and human monocytes (U-937, CRL-1593.2, American Type Culture Collection, RRID:CVCL_0007). Injury and treatment models were fitted to cell type based on previous studies.

HK-2 cells were seeded at a density of 0.8 × 10^6^ cells/T25 flask. 24 h later, HK2 cells were divided into three groups: Group 1 cells were cultured under normal conditions (non-injured), while Groups 2 and 3 cells were pre-treated with high glucose (HG, 25 mmol/L, 50-99-7, Sigma–Aldrich) and TGFβ1 (5 ng/ml, 240-B-002, R&D Systems) for 6 h, and Group 3 cells were subsequently treated with D (50 nM) and Q (5 μM), then incubated 18 additional hours. Afterwards, all HK2 cells were lysed and prepared for qPCR analyses.

HUVECs were seeded at a density of 0.8 × 10^6^ cells/T25 flask. HUVECs at earlier and late passages including p4 and p9 were divided into five groups for experimentation: Group 1 (p4), Group 4 (p9), and Group 5 (p9) cells were cultured under normal conditions (non-injured), while Group 2 (p4) and 3 (p4) cells were pre-treated with HG (25 mmol/L) and TNF (20 ng/ml) for 6 h, and Groups 3 and 5 cells were subsequently treated with D (100 nM) and Q (10 μM), then incubated for 18 additional hours. Subsequently, all HUVEC were lysed and prepared for qPCR. Groups 1, 2, and 3 and Groups 1, 4, and 5 were compared separately.

U-937 cells were grown and maintained in a complete medium. To induce differentiation into macrophages (Mϕ), U-937 cells (1.5 × 10^6^ cells/T25 flask) were cultured in RPMI-1640 medium with 100 ng/ml PMA (Sigma–Aldrich, P8139) overnight. Afterwards, the medium was removed, and cells were washed with PBS once. Mϕ were divided into three groups: Group 1 was cultured under normal conditions (non-injured), while Groups 2 and 3 were pre-treated with HG (25 mmol/L) and LPS (100 ng/ml, L2018 Sigma–Aldrich) for 6 h, and Group 3 cells were subsequently treated with D (200 nM) and Q (20 μM), then incubated for 40 additional hours. Afterwards, all Mϕ were lysed and prepared for quantitative real-time polymerase chain reaction (qPCR) and immunofluorescence (IF) analyses.

### qPCR

Total RNA was isolated from kidney tissue (0.5–0.05 g) using PureLink RNA Mini Kit (Invitrogen, 12183018A, Waltham, MA) and cDNA was synthesised using SuperScript VILO Master Mix (Invitrogen, 11755050). Gene expression was analysed by qPCR using Applied Biosystems QuantStudio Real-Time PCR software (Life Technologies, Carlsbad, CA). Target mRNAs are grouped into functional categories and include the following: Kidney injury markers: *LGALS3* (encoding galectin-3), *HAVCR1* (encoding KIM-1), *TNFRSF1A* and *TNFRSF1B* (encoding TNFR 1 and TNFR2). Fibrosis and epithelial–mesenchymal transition (EMT) markers: *TGFB1*, *FN1* *(encoding fibronectin)*, *COL1A1*, *CDH1* (encoding E-cadherin), *TNC* (encoding Tenascin-C), and *INHBA* (encoding Activin A, also a SASP marker). Inflammatory mediators and macrophages phenotypes: *IL1B*, *IL6*, *CD206*, *CD86*, *CD38*, *ADGRE1* (encoding F4/80), *CCL2* (encoding MCP-1), *TNF* (encoding TNF-α), *NFKB1* (encoding p50 subunit), and *EGR2* (encoding early growth response protein 2). Senescence markers: *CDKN2D* (encoding p19), *CDKN2A* (encoding p16), *CDKN1A* (encoding p21). Geroprotective factors: *SIRT1* (encoding sirtuin-1), and *KL* (encoding α-Klotho). Gene expression was normalised to *GAPDH* or TATA-box binding protein (*TBP*). The 2^−ΔΔCT^ method was used to calculate fold-change of gene expression (See Supplemental Methods).

### IF

HK-2 cells, HUVECs, and macrophages were plated in 24-well plates and stained using primary antibodies diluted 1:100 against NF-κB p65 (ab32536, RRID:AB_776751), p16 INK4A (ab189034, RRID:AB_2737282), and Activin A (PA5-100101, RRID:AB_2815631), and secondary antibody Goat anti-Rabbit IgG (H + L) Alexa Fluor 594 (A-11012, RRID:AB_2534079). Analysis was performed by imaging using EVOS M5000 (Invitrogen, Waltham, MA) and the percent of positive staining was calculated using ImageJ software by counting positively stained cells and normalising to DAPI nuclei stain (See Supplemental Methods).

### Statistical analyses

Statistical analysis was performed using GraphPad Prism 9. Data distribution was evaluated using the D'Agostino-Pearson normality test. Normally distributed data were expressed as mean ± SEM. Parametric tests (ANOVA and t-tests) were used as appropriate. All data were considered significant if p ≤ 0.05.

### Role of funders

Funders had no role in study design, data collection, data analyses, interpretation, or writing of the report.

## Results

### D + Q improves kidney function and reduces kidney injury in murine DKD

As per model design, fasting blood glucose and kidney-to-body weight ratio in STZ mice were higher versus Controls (Fig. 1C and Figure S1A). On histology, higher glomerular injury scores, primarily based on degree of mesangial matrix expansion, were identified in STZ mice versus Control. Similarly, podocyte marker nephrin was reduced in STZ mice versus Control (Fig. 1B). Kidney function was lower (higher plasma creatinine) in the STZ groups. Other markers of kidney injury and predictive markers of DKD progression were impacted by STZ, including decreased urinary epidermal growth factor (EGF),^45^ increased 24 h urinary albumin, and increased kidney mRNA expression *of LGALS3* (Mac-2),^46^ *HAVCR1 (KIM-1)*, *TNFRSF1B (TNFR2)*^47^ *and TNFRSF1A (TNFR1)* versus Control (Fig. 1C and D, Figure S1A and B). D + Q-treated mice had lower mRNA expression of *LGALS3* with higher urine EGF, lower glomerular injury scores, and lower creatinine (indicating improved kidney function) versus STZ. Non-significant trends were observed with lower *HAVCR1* and *TNFRSF1B,* and increased nephrin staining. Contrarily, fasting glucose levels did not differ between D + Q treated and STZ mice, suggesting that the effects were unrelated to glucose control.

### D + Q reduces fibrosis and extracellular matrix (ECM) deposition in DKD

**Fig. 2:**
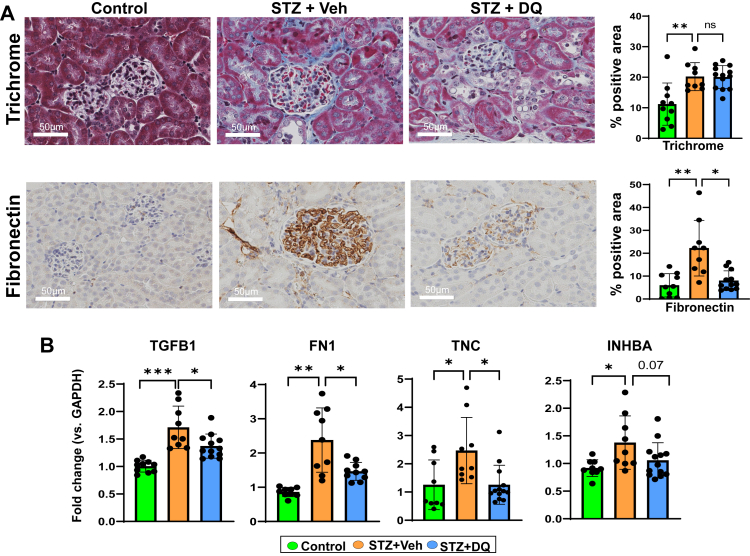
**D + Q reduces diabetic kidney fibrosis and extracellular matrix protein deposition. A)** Representative images of Masson's Trichrome staining and fibronectin immunohistochemistry of paraffin-embedded kidney sections (Scale bars = 50 μm). Quantification of fibrosis (% blue area of Masson's Trichrome staining) and FN immunohistochemistry (% brown area). **B)** qRT-PCR of kidney tissue for fibrosis related factors: *TGFB1*, *FN1*, TNC, and *INHBA*. All data are shown as mean ± SEM. ∗p < 0.05, ∗∗p < 0.01, ∗∗∗p < 0.005, and ∗∗∗∗p < 0.001. Data were analysed with unpaired t-tests or ANOVA where appropriate. D + Q: dasatinib plus quercetin, FN1: fibronectin 1, TGFB1: transforming growth factor beta 1, TNC: tenascin C, INHBA: inhibin subunit beta A.

Kidney fibrosis (trichrome), pro-fibrotic cytokines (TGFβ1 and activin A, which is also a SASP marker), and components or mediators of ECM (fibronectin and tenascin-C) were examined (Fig. 2). STZ mice had increased trichrome and fibronectin staining versus Controls. Similarly, mRNA expression of *TGFB1*, *FN1*, *TNC*, and *INHBA* were increased in this group. D + Q led to lower fibronectin staining, but not trichrome. Similarly, D + Q-treated mice had lower mRNA expression of *TGFB1*, *FN1*, *TNC*, and trended lower for *INHBA* versus the STZ group.

### D + Q reduces inflammatory mediators or SASP in DKD

**Fig. 3:**
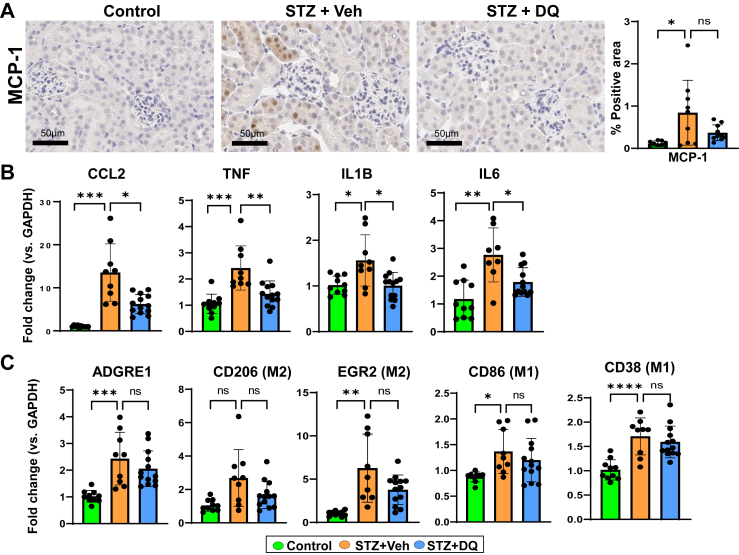
**D + Q reduces inflammatory mediators (and the senescence-associated secretory phenotype) in the diabetic kidney.** A) Representative images and quantification of immunohistochemistry (% brown area) of paraffin embedded kidney sections for MCP-1 (Scale bar = 50 µm). B) qRT-PCR of kidney tissue for inflammation markers: CCL2, TNF, IL-1β, and IL-6. C) Markers of macrophages (M2 and M1): ADGRE1 (M1 and M2), CD206 (M2), EGR2 (M2), CD38 (M1), CD86 (M1). All data are shown as mean ± SEM. ∗p < 0.05, ∗∗p < 0.01, ∗∗∗p < 0.005, and ∗∗∗∗p < 0.001. Data were analyzed with unpaired t-tests or ANOVA where appropriate. D + Q: dasatinib plus quercetin, MCP-1: monocyte chemoattractant protein, CCL2: C–C motif chemokine ligand 2, TNF: tumor necrosis factor-α, IL-1β: interleukin-1β, IL-6: interleukin-6, ADGRE1: adhesion G protein-coupled receptor E1 (F4/80), EGR2: early growth response protein 2, STZ: streptozotocin.

In STZ mice, MCP-1 (CCL2) staining and mRNA expression was increased versus Controls (Fig. 3A and B). Similarly, inflammatory cytokines or SASP components (*TNF, IL-1B*, *IL6*) mRNA expression was higher. Macrophage marker (F4/80; *ADGRE1*) was increased in STZ, and both M1-like (CD38 and CD86) and M2-like (*EGR2*) macrophages phenotypes increased (Fig. 3C). D + Q administration led to lower mRNA expression of inflammatory mediators versus STZ. However, D + Q did not significantly reduce macrophage (*ADGRE1*) abundance in the kidney, despite a reduction in MCP-1. Interestingly, NF-κB (*NFKB1*) was unaffected by STZ or D + Q therapy (Figure S1C). CD45 (*PTPRC*) was not different across mice groups (Figure S1B).

### D + Q decreases senescent cell burden and restores geroprotective factors in DKD

**Fig. 4:**
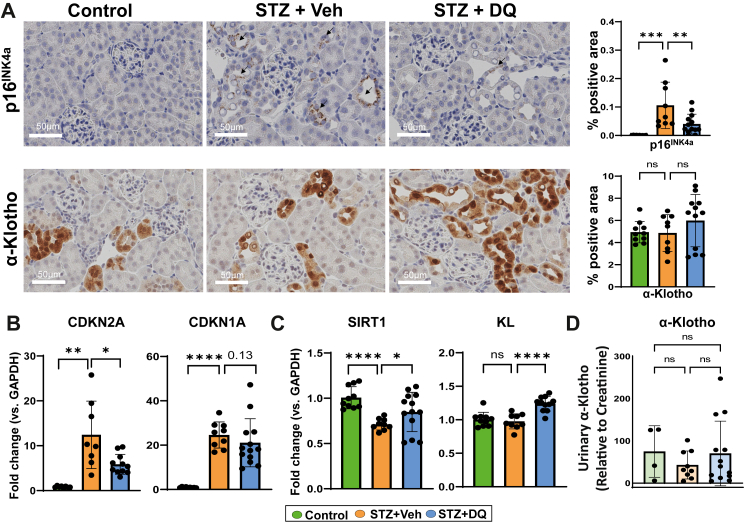
**D + Q reduces diabetic kidney senescent cell burden and restores geroprotective factors. A)** Representative images and quantification of immunohistochemistry (% brown area) of paraffin-embedded kidney sections for p16 and α-Klotho (Scale bar = 50 µm). **B)** qRT-PCR of kidney tissue for markers of senescent cell burden, *CDKN2A* and *CDKN1A*. **C)** Geroprotective markers, *SIRT1* and *KL*. **D)** ELISA for urine α-Klotho normalized to creatinine levels. All data are shown as mean ± SEM. ∗p < 0.05, ∗∗p < 0.01, ∗∗∗p < 0.005, and ∗∗∗∗p < 0.001. Data were analysed with unpaired t-tests or ANOVA where appropriate. D + Q: dasatinib plus quercetin, CDKN2A: cyclin dependent kinase inhibitor 2A (p16^INK4a^); CDKN1A: cyclin dependent kinase inhibitor 1A (p21^CIP1^); SIRT1: sirtuin 1, KL: Klotho.

In STZ mice, the senescence markers p16 and p21 (protein and mRNA) were higher versus Controls (Fig. 4A and B and Figure S1E). p19 trended similarly but did not reach significance (Figure S1D). The senescence marker SA-β-gal activity was increased in STZ versus Controls (Figure S1E). While the geroprotective factor SIRT1 was decreased in STZ, α-Klotho (*KL*) expression was not different from Controls at either the protein or mRNA levels. D + Q administration led to lower senescence abundance (p16 and trended towards lower p21) and higher geroprotective factors (SIRT1 and α-Klotho) versus STZ (Fig. 4C). Protein expression of urinary α-Klotho was not different among groups (Fig. 4D).

### D + Q reduces injury in HK2, macrophages, and HUVECs exposed to HG *in vitro*

To explore senolytic effects in human cells exposed to a diabetic *milieu*, HK2 cells (HG + TGFβ1), HUVECs (HG + TNF), and macrophages (HG + LPS) were studied.

**Fig. 5:**
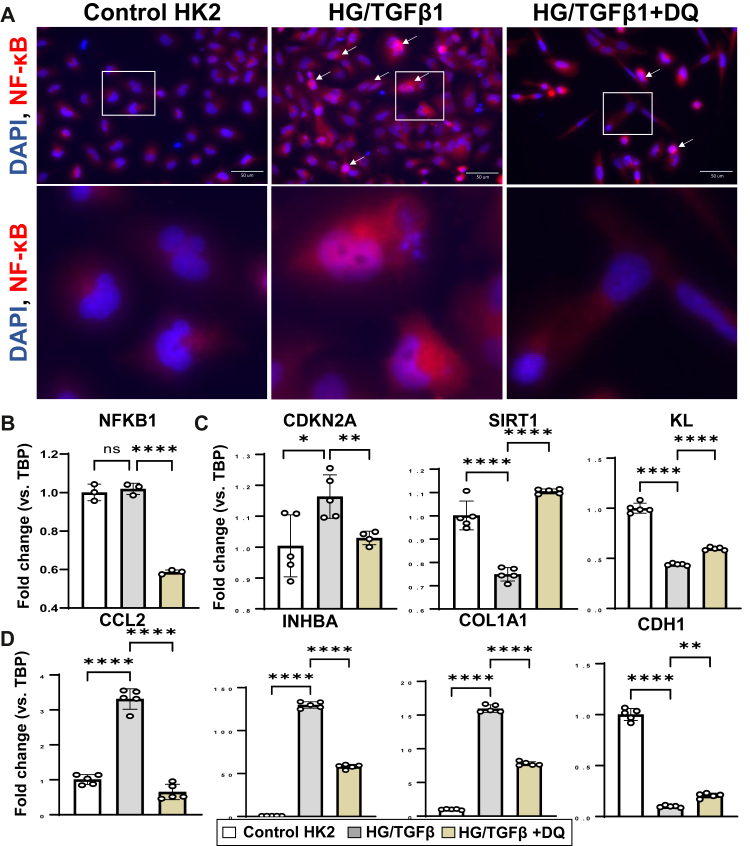
**D + Q restores geroprotective factors and reduces pro-inflammatory and pro-fibrotic factors in HK2 cells.** HK2 were pretreated with high glucose (25 mM) & TGFβ1 (5 ng/ml) 6 h and subsequently with dasatinib (50 nM) and quercetin (5 μM) 18 h. **A)** Representative images of immunofluorescent staining of NF-κB (p65) with zoomed sections to highlight nuclear translocation. qRT-PCR for **B)** NFKB1, **C)** senescence marker *CDKN2A* and geroprotective factors *SIRT1* and *KL*, **D)** pro-inflammatory and pro-fibrotic factors *CCL2*, *INHBA*, *COL1A1*, and *CDH1*. All data are shown as mean ± SEM. ∗p < 0.05, ∗∗p < 0.01, ∗∗∗p < 0.005, and ∗∗∗∗p < 0.001. Data were analysed with unpaired t-tests or ANOVA where appropriate. D + Q: dasatinib plus quercetin, TGFB1: transforming growth factor beta 1, NFKB1: nuclear factor kappa B subunit 1, CDKN2A: cyclin dependent kinase inhibitor 2A (p16^INK4a^), SIRT1: sirtuin 1, KL: Klotho, CCL2: C-C motif chemokine ligand 2 (MCP-1), INHBA: inhibin subunit beta A (activin A), COL1A1: collagen type I alpha 1 chain, CDH1: cadherin 1.

In HK2 cells, HG + TGFβ1 induced the robust activation of the NF-κB signalling pathway, as demonstrated by *p65* nuclear translocation and upregulation of *CCL2* and *INHBA* (Fig. 5 and Figure S3A). This was accompanied by the concomitant activation of cellular senescence pathways, evidenced by reduced levels of *KL* and *SIRT 1*, alongside increased p16 mRNA expression. Consequently, a key phenotypic transition toward fibrosis was observed, characterised by elevated *COL1A1* and decreased *CDH1*. Notably, D + Q treatment effectively mitigated the HG + TGF-β1-induced cellular senescence, suppressed activation of NF-κB signalling, and attenuated the fibrotic phenotype. Total cell count was reduced, but viability was preserved after D + Q treatment (Figure S3B).

**Fig. 6:**
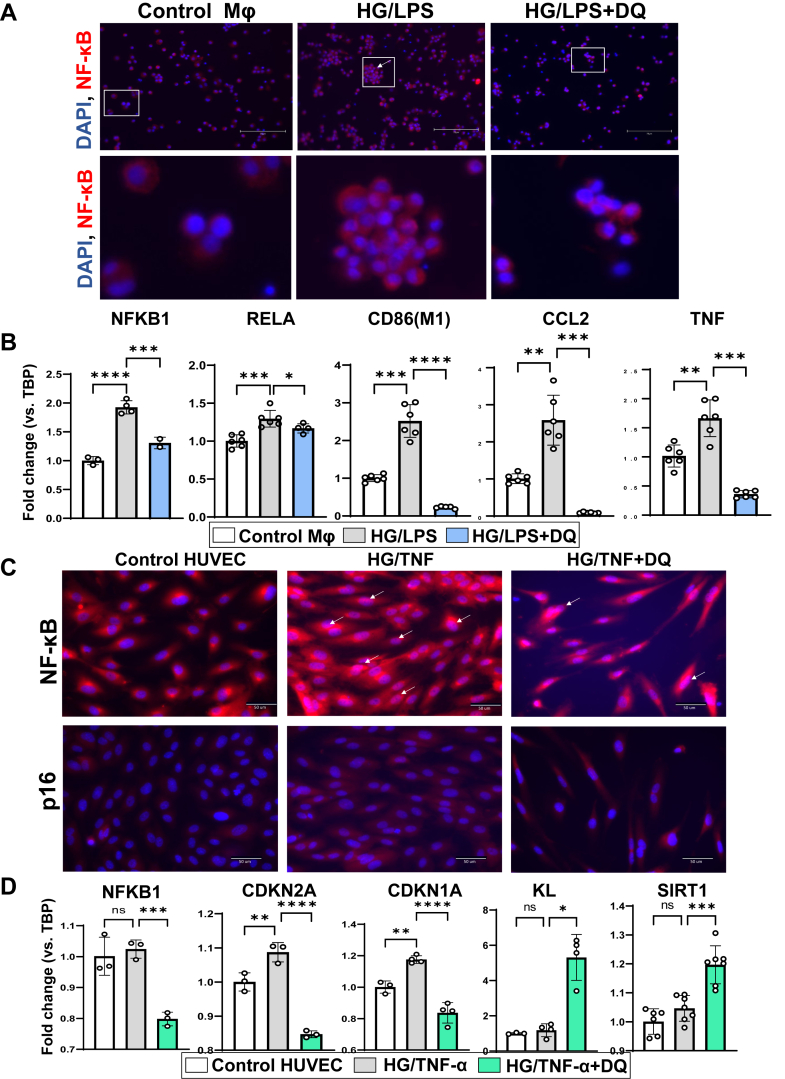
**D + Q removes senescent cells and preserves geroprotective factors in HUVECs**, **inhibits polarization to M1 and pro-inflammatory factors in macrophages.** U937 cells were treated with PMA overnight to induce switching into macrophages (Mϕ). Mϕ were pretreated with high glucose (25 mM) & LPS (100 ng/ml) 6 h and subsequently with dasatinib (200 nM) and quercetin (20 μM) 40 h. HUVEC were pretreated with high glucose (25 mM) & TNF (20 ng/ml) 6 h and were subsequently treated with dasatinib (100 nM) and quercetin (10 μM) 18 h. **A)** Representative images of immunofluorescent staining for NF-kB (p65) in U937 macrophages, with zoomed images for better clarity. **B)** qRT-PCR of macrophages for *NFKB1, RELA,* for *CD86* (M1-like phenotype), *CCL2*, and *TNF*. **C)** Representative images of immunofluorescent staining for NF-kB (p65) and p16 in HUVEC. **D)** qRT-PCR of HUVEC for *NFKB1*, *CDKN2A*, *CDKN1A*, KL, and *SIRT1*. All data are shown as mean ± SEM. ∗p < 0.05, ∗∗p < 0.01, ∗∗∗p < 0.005, and ∗∗∗∗p < 0.001. Data were analysed with unpaired t-tests or ANOVA where appropriate. D + Q: dasatinib plus quercetin, PMA: Phorbol 12-myristate 13-acetate, LPS: Lipopolysaccharide, NFKB1: nuclear factor kappa B subunit 1, CCL2: C-C motif chemokine ligand 2 (MCP-1), TNF: Tumor Necrosis Factor, CDKN2A: cyclin dependent kinase inhibitor 2A (p16^INK4a^); CDKN1A: cyclin dependent kinase inhibitor 1A (p21^CIP1^); SIRT1: sirtuin 1, KL: Klotho.

HG + LPS induced macrophage nuclear translocation of NF-κB p65 and significantly upregulated the mRNA expression of the *NFKB1*, which encodes the p50 subunit. This NF-κB pathway activation was effectively suppressed at both the mRNA and protein levels by clearing senescent cells with D + Q treatment (Fig. 6A). Furthermore, HG + LPS promoted the polarisation of monocytes toward an M1-like phenotype, as indicated by the marker CD86. D + Q treatment not only inhibited this inflammatory polarisation but also downregulated the expression of the key pro-inflammatory genes *CCL2* and *TNF* (Fig. 6B).

HUVECs (passage 4, P4) exposed to HG + TNF had increased p16 and p21 mRNAs expression (Fig. 6C and D). Senescent cells were selectively removed after D + Q treatment. The geroprotective factors, α-Klotho and SIRT1, did not differ between HG + TNF versus controls, but increased with D + Q treatment. Gene expression of *NFKB1* p50 and *RELA* p65, and nuclear translocation of NF-κB p65 were reduced after D + Q. Replicative senescent HUVECs at a higher passage number (passage 9, P9) exhibited increased p16 (CDKN mRNA expression versus P4 and p16, which decreased significantly after D + Q treatment while α-Klotho had a reversed pattern (Figure S2).

## Discussion

Our major findings include the following: 1) A 5-day oral gavage regimen of D + Q senolytic therapy reduced senescent cell abundance, improved kidney function and injury markers, and reduced ECM remodelling and fibrosis. 2) Cellular senescence, characterised by upregulation of p16, p21, several SASP markers, and SA-β-gal activity, was increased in this STZ-induced DKD model and decreased after D + Q treatment. 3) In human cells (macrophages, endothelial cells, and renal epithelial cells) *in vitro*, D + Q reduced senescent cell abundance, increased the geroprotective factors, α-Klotho and SIRT1, and inhibited NF-κB pathway activation after hyperglyceamic exposure. Collectively, our findings provide translational evidence that strategic senolytic intervention with D + Q not only removes pathogenic senescent cells and their paracrine, proinflammatory activities but also reinvigorates intrinsic anti-ageing defences, thereby showing promise as a therapeutic for DKD.

Pathogenic mechanisms of senescent cells include paracrine signalling through their SASP, which promotes inflammation and contributes to disease progression under several conditions.^9^^,^^23^^,^^48^ In our previous pilot clinical trial with DKD participants, we found that a 3-day oral D + Q course decreased adipose and epidermal senescent cell abundance 11 days later, with a reduction in p16 and p21-positive expressing cells, SA-β-gal activity, and key circulating SASP factors, including IL-1α, IL-6, and MMPs.^32^ The current study in a DKD animal model now demonstrates that a 5-day oral gavage D + Q regimen has reparative effects in the diabetic kidney with decreased senescent cell abundance and SASP factors (IL-1β, IL-6, TNF, TGFβ1, and activin A) after 20 days. In line with these observations, our *in vitro* studies showed that HK2 cells and HUVECs in a diabetic *milieu* develop increased senescence markers (p16 and p21) that are reduced after D + Q treatment.

To confirm the protective effects of D + Q in our DKD model, we investigated changes in kidney function, histology (glomerular injury score), fibrosis and the ECM, markers of kidney injury (KIM-1), and predictors of DKD progression (TNFR1, TNFR2,^47^ Galectin-3)^49^, ^50^, ^51^ and urine EGF. Urine EGF levels predicted rapid decline in kidney function in three independent patient cohorts with moderately advanced CKD^52^ and over 600 patients with Type-2 diabetes with preserved kidney function.^45^ These findings are consistent with a focal reduction in EGF expression in atrophic and injured (KIM-1^+^) tubules in a diabetic and hypertensive mouse model.^45^ In a large Type-2 diabetes cohort without albuminuria or reduced kidney function, low urinary EGF concentration was associated with an increased risk of rapid loss of kidney function independently from classic risk factors.^45^ In our study, D + Q improved histological lesions (ECM, fibrosis, glomerular injury), kidney function, and the DKD progression marker Galectin-3 (showing trends for KIM-1, TNFR1, TNFR2 reduction). Furthermore, D + Q improved urine EGF levels in diabetic mice to those of healthy controls, further confirming restorative activities. Chronic inflammation is a major contributor to DKD pathogenesis and macrophages are the major infiltrating inflammatory cells in human and mouse diabetic kidneys.^53^ Activated macrophages propagate SASP factors in senescent tissues through pathways including NF-κB that further contribute to recruitment and activation of macrophages.^9^^,^^54^, ^55^, ^56^ Hence, targeting macrophage activation and monocyte-recruiting chemokines has been extensively investigated in preclinical and clinical DKD studies.^57^, ^58^, ^59^, ^60^ In this study, diabetes increased kidney MCP-1, TNF, IL1B, and macrophage abundance. D + Q significantly reduced MCP-1, TNF, IL1B, and IL-6. Interestingly, D + Q inhibited NF-κB translocation and MCP-1 and TNF expression in macrophages activated by HG + LPS exposure *in vitro*, while non-significant trends were observed for reduction in kidney NF-κB expression with the current therapeutic regimen. We postulate that the sustained NF-κB activation relies predominantly on the recurrent utilisation of pre-existing, cytoplasmic pools of p50–p65 dimers, without engaging the strong positive feedback loop for NFKB1 gene transcription. We also found that D + Q decreased senescent cells and inhibited the SASP at the time of kidney harvest. In addition, non-significant trends were observed for reduction in kidney macrophage (F4/80) and M1/M2-like phenotype abundance. However, the MCP-1 mRNA and pro-inflammatory M1-like phenotype (CD86) were decreased after senescent cell clearance *in vitro*. In our prior pilot DKD clinical trial, we observed that D + Q reduced CD68+ macrophage abundance in abdominal subcutaneous adipose tissue.^32^ In obese mice, pre-treating with D + Q attenuated trafficking of labelled monocytes into adipose tissue.^2^ Hence, it is not unlikely that macrophage numbers were decreased by a reduction in macrophage attractants and anchoring by senescent adipose cells, instead of directly removing senescent macrophages.^28^ Hence, D + Q may limit attraction of macrophages in the diabetic kidney microenvironment.

The effect of D + Q on geroprotective factors in the current study should also be highlighted. Under physiological conditions, the anti-ageing proteins α-Klotho and SIRT1 can protect against kidney ageing,^61^^,^^62^ but are downregulated under diabetic conditions, which may accelerate kidney ageing and injury in DKD.^14^^,^^63^, ^64^, ^65^ Similar to other studies,^66^^,^^67^ α-Klotho appears more abundant in kidney tubules than glomeruli. In other STZ-induced diabetic mouse studies, renal α-Klotho mRNA expression levels were decreased significantly 6–9 weeks after diabetes induction.^68^, ^69^, ^70^ Surprisingly, we found no noticeable difference in α-Klotho gene or protein expression between control and diabetic mice at 15–16 weeks. These findings may be related to biological variations between the DKD models, different stages of kidney progression, and compensatory elevation that antagonises kidney tubular injury. Notably, D + Q increased α-Klotho mRNA expression to levels above diabetic and control groups. *In vitro* studies confirmed improvement in α-Klotho mRNA expression after D + Q exposure in HK2 and HUVECs. We also found that D + Q can improve SIRT1 mRNA expression *in vivo* and *in vitro* in a diabetic *milieu*. In normal kidneys, SIRT1 is widely expressed in tubular cells, glomerular endothelial cells, and podocytes, and exerts significant renoprotective effects by inhibiting cell apoptosis, inflammation, and fibrosis.^63^^,^^65^^,^^71^ Conditional deletion of SIRT1 in podocytes of Type 2 diabetic (db/db) mice resulted in the acetylation of the p65 subunit of NF-κB and STAT3, increased proteinuria, and led to more severe kidney damage.^72^ Moreover, SIRT1 activators substantially attenuated kidney fibrosis processes through deacetylation of Smad3.^73^^,^^74^ Our study identified a reduction in SIRT1 mRNA expression in diabetic kidneys versus controls which was restored with D + Q oral administration. In culture, D + Q restored SIRT1 expression to levels higher than untreated HK2 controls. While no difference was found between controls and HG + TGFβ-treated HUVECs, D + Q led to higher SIRT1 mRNA expression versus controls. Collectively, α-Klotho and SIRT1 hold protective mechanisms that are restored with D + Q therapy in DKD.

While providing unique insights, our study has limitations. Cellular senescence characterisation may be unique to the disease or condition and associated inducers of senescent state. This study primarily relies on three of potential 8 hallmarks available as described by the SenNet working group.^15^ However, recent senescence biomarker developments may enhance future investigations in kidney senescence, particularly in diabetes.^5^ While kidney tissue studies were revealing, our finding of unchanged urinary α-Klotho among the groups was unanticipated. α-Klotho is a temperature-sensitive protein which may degrade during standardised 24 h collections and subsequent storage. Additional methods may be needed to optimise investigations in DKD mice where urine α-Klotho may be low and hence preclude reliable intergroup comparisons. Finally, conducting these studies in additional DKD models will increase generalisability and enhance the ability to determine treatment effect and/or mechanism differences between diabetes types.

### Conclusions

Our findings demonstrate the therapeutic effects of senolytic agents, D + Q, in a murine model of DKD. Notably, “hit-and-run” treatment improved kidney function and mitigated murine DKD injury by modulating the inflammatory landscape, reducing senescent cell abundance, and restoring geroprotective factors. This supports the growing body of literature on senescence and senotherapeutics in preclinical and clinical DKD investigations. Encouragingly, further D + Q clinical investigations that optimise this therapeutic strategy may lead to increased health-span in individuals with DKD.

## Contributors

XHB, JLK, LOL, TT, YZ, SK, SRK and LJH contributed conception and design of the study. XHB, ZKS, ALB, CJZ, KME, JLK, LOL, TT, YZ, SK, SRK and LJH collected the data and contributed to data analysis and interpretation. LJH acquired the study samples and/or research resources/materials. XHB, ZKS, ALB, CJZ, KME, LJH accessed and verified the data. LJH supervised the study. XHB wrote the first draft of the manuscript and all other authors reviewed and made edits to the manuscript. LJH provided financial support. All authors have edited and agreed to the published version of the manuscript.

## Data sharing statement

The datasets supporting the conclusions of this article are available from the corresponding author on reasonable request. Data requests through email will be shared with a signed data access agreement or other arrangements based upon approved and agreed upon study plan.

## Declaration of interests

LJH serves as a consultant for Resolution Therapeutics and ETTA Biotechnology. LOL is an advisor to CureSpec, Ribocure Pharmaceuticals, Cellergy, and LiveKidney.bio. JLK and TT have a financial interest related to this research. Patents on the senolytic drugs discovered by JLK and TT are held by Mayo Clinic. JLK is an AFAR board member and HLMS member. This research was conducted in compliance with Mayo Clinic Conflict of Interest policies. No conflicts of interest, financial or otherwise, are declared by the other authors.

## Appendix A Supplementary data

Supplementary Material Figure S1. Comparisons between control and diabetic (STZ-induced) mice with and without D+Q. **A)** Endpoint body weight and kidney-to-body weight ratio, and ELISA of urine albumin normalized to creatinine. **B)** qRT-PCR of kidney tissue for *TNFRSF1A*, *NFKB1,* and *PTPRC*. **C)** qRT-PCR for senescence marker *CDKN2D* and kidney ELISA for geroprotective factor *SIRT1*. E**)** Representative images are shown for senescence-associated β-galactosidase and p21 staining. All data are shown as mean ± SEM. ∗ p<0.05, ∗∗ p<0.01, and ∗∗∗ p<0.005. STZ: streptozotocin, D+Q: dasatinib plus quercetin, *TNFRSF1A:* tumor necrosis factor receptor superfamily member 1A (for TNFR1*),* NFKB1: nuclear factor kappa B subunit 1, CDKN2D: cyclin dependent kinase inhibitor 2D (for p19), SIRT1: sirtuin 1, PTPRC: Protein tyrosine phosphatase, receptor type, C (for CD45). Figure S2. HUVEC (passage 4 and passage 9) were pre-treated with high glucose (25 mM) & TNF (20 ng/mL) 6 h and were subsequently treated with dasatinib (100 nM) and quercetin(10 μM) 18 h. **A)** qRT-PCR for p16 and α-Klotho. **B)** Representative images of immunofluorescent staining for NF-κB (p65), with zoomed sections to highlight nuclear translocation. All data are shown as mean ± SEM. ∗ p<0.05, ∗∗ p<0.01, and ∗∗∗ p<0.005. D+Q: dasatinib plus quercetin, HUVEC: human umbilical vein endothelial cells, TNF: tumor necrosis factor. Figure S3. HK2 were pre-treated with high glucose (25 mM) & TGFβ1 (5 ng/ml) 6 h and subsequently with dasatinib (50 nM) and quercetin (5 μM) 18 h. **A)** Western blot results for NF-κB and housekeeper β-actin. **B)** Trypan Blue total cell counts and viability results.
