# Supplementary material for: Senloytics, dasatinib plus quercetin, reduce kidney inflammation, senescent cell abundance, and injury while restoring geroprotective factors in murine diabetic kidney disease
Source: eBioMedicine. 2026 Jan 20;124:106124. doi: 10.1016/j.ebiom.2026.106124 (PMC12857402; doi:10.1016/j.ebiom.2026.106124)
Supplement: Supplementary Material — Figure S1. Comparisons between control and diabetic (STZ-induced) mice with and without D+Q. A) Endpoint body weight and kidney-to-body weight ratio, and ELISA of urine albumin normalized to creatinine. B) qRT-PCR of kidney tissue for TNFRSF1A, NFKB1, and PTPRC. C) qRT-PCR for senescence marker CDKN2D and kidney ELISA for geroprotective factor SIRT1. E) Representative images are shown for senescence-associated β-galactosidase and p21 staining. All data are shown as mean ± SEM. ∗ p<0.05, ∗∗ p<0.01, and ∗∗∗ p<0.005. STZ: streptozotocin, D+Q: dasatinib plus quercetin, TNFRSF1A: tumor necrosis factor receptor superfamily member 1A (for TNFR1), NFKB1: nuclear factor kappa B subunit 1, CDKN2D: cyclin dependent kinase inhibitor 2D (for p19), SIRT1: sirtuin 1, PTPRC: Protein tyrosine phosphatase, receptor type, C (for CD45). Figure S2. HUVEC (passage 4 and passage 9) were pre-treated with high glucose (25 mM) & TNF (20 ng/mL) 6 h and were subsequently treated with dasatinib (100 nM) and quercetin(10 μM) 18 h. A) qRT-PCR for p16 and α-Klotho. B) Representative images of immunofluorescent staining for NF-κB (p65), with zoomed sections to highlight nuclear translocation. All data are shown as mean ± SEM. ∗ p<0.05, ∗∗ p<0.01, and ∗∗∗ p<0.005. D+Q: dasatinib plus quercetin, HUVEC: human umbilical vein endothelial cells, TNF: tumor necrosis factor. Figure S3. HK2 were pre-treated with high glucose (25 mM) & TGFβ1 (5 ng/ml) 6 h and subsequently with dasatinib (50 nM) and quercetin (5 μM) 18 h. A) Western blot results for NF-κB and housekeeper β-actin. B) Trypan Blue total cell counts and viability results. [file mmc1.pdf]

Supplemental Materials

Detailed Methods
Supplemental Figure S1
Supplemental Figure S2
Supplemental Figure S3

Methods

Mice

All animal experiments were approved by the Mayo Clinic Institutional Animal Care and Use Committee. Mice were housed 3-5/cage under standard laboratory conditions: 21-23°C, 50% humidity, 12h light-dark cycle, municipal city water, and food ad libitum.

Immunofluorescence

HK-2 cells, HUVEC, and macrophages were plated in 24-well plates and stained using primary antibodies, NF-KB (ab32536), p16 INK4A (ab189034), and Activin A (PA5-100101), and secondary antibody Goat anti-Rabbit IgG (H+L) Alexa Fluor 594 (A-11012). Antibodies were diluted 1:100 in 1% BSA+ 3% Triton. Cells were fixed using 4% formaldehyde for 20 minutes at room temperature. The wells were washed twice with PBS and then permeabilized with 0·2% Triton for 5 minutes. Cells were washed three times and blocked with 5% BSA + 0·2% Triton for 30 minutes at room temperature. Cells were stained with primary antibody and incubated overnight at 4°C. After overnight incubation, cells were rinsed three times and stained with secondary antibodies in a dark room at room temperature for 30 minutes. The wells were washed three times and then counterstained with DAPI (P36941). Analysis was performed by imaging wells using EVOS M5000 (Invitrogen, Waltham, MA), and the percent of positive staining was calculated using ImageJ software by counting instances of positively stained cells and normalized to DAPI nuclei stain.

Histology and immunohistochemistry

Kidney tissue was fixed with 10% formalin, embedded in paraffin, stained with PAS or Trichrome reagents, and examined under an optical microscope. The glomerular injury score was calculated in PAS sections as previously described.43 In brief, the score was assessed on a 0-4 scale (0 = normal, 1 = mild mesangial matrix expansion, 2 = moderate matrix expansion with patent capillaries, 3 = severe matrix expansion with segmental capillary loop consolidation, 4 = severe matrix expansion with global capillary loop consolidation), ≥10 random fields taken at 20× objective per kidney were examined and analyzed. Expression of Nephrin, (1:500, ab216341, Abcam), Fibronectin (1:2500, ab268020, Abcam), p16 (1:100, ab189034, Abcam), and α-Klotho (1:400, MA5-32784, Invitrogen) were examined on paraffin-embedded renal tissue sections (5 mm) by IHC analysis. Each section was captured for ≥10 images randomly. Digital pictures were taken using the Aperio AT2 Scanner and the Image Scope software with a 20x and 40x objective. The percent area stained of the total tissue area was calculated using MATLAB and Aperio analysis software.

Senescence-associated beta-galactosidase Staining

Frozen kidney tissue was sliced using a cryostat slicer, mounted on microscope slides, and were then fixed and stained for Senescence β-Galactosidase (Cell Signalling Technology, 9860), according to manufacturer protocol.

Real-time PCR Probes

All probes were obtained from Life Technologies, Carlsbad, CA: Activin A (Mm00434339_m1, Hs01081598_m1), AIM2 (Mm01295719_m1), CCL-22 (Mm00436439_m1), CD38 (Mm00483143_m1), CD45 (Mm01293577_m1), CD86 (Mm01344638_m1, HS00199349_m1), CD206 (Mm00485148_m1), CDH1 (HS01023895_m1), Col1A1 (Mm00801666_g1, Hs00164004_m1), CTGF (Mm01192933_g1), Egr2 (Mm00456650_m1), F4/80 (Mm00802529_m1), FN1 (Mm01256744_m1), GAPDH (Mm99999915_g1), IL-1β (Mm00434228_m1), IL-6 (Mm00446190_m1), IL-10 (Mm01288386_m1), KIM1 (Mm00506686_m1), Klotho (Mm00502002_m1, Hs00934627_m1), Lmnb1 (Mm00521949_m1), Mac-2 (Mm00802901_m1), MCP1 (Mm00441242_m1, Hs00234140_m1), NFkb1 (Mm00476361_m1, Hs00765730_m1), p16 (Mm00494449_m1, Hs00923894_m1), P19 (Mm00486943_m1), P21 (Mm00432448_m1), P53 (Mm01731290_g1), RELA (Hs01042014_m1), Rgn (Mm00485711_m1), Sirt1 (Mm01168521_m1, Hs01009006_m1), TBP (Hs00427620_m1), TGF-β1, (Mm01178820_m1), TNFα (Mm00443258_m1, Hs00174128_m1), TNF-R1 (Mm00441881_g1). The $2^{-\Delta\Delta CT}$ method was used to calculate the fold-change of gene expression.

Western Blotting

HK-2 cells were lysed in Pierce RIPA buffer (89900, Thermo Scientific). Samples were centrifuged at 14,000 g at 4°C for 20 minutes, and supernatant was collected for protein quantification using Pierce Rapid Gold BCA Protein Assay Kit (A53226, Thermo Scientific). Samples were prepared with lithium dodecyl sulfate sample buffer (84788, Thermo Scientific), and 10µg protein were loaded on 4–20% Mini-PROTEAN® TGX™ Precast Protein Gels (4561095, Bio-Rad) and separated by electrophoresis. After transferring using the Power Blotter System (Invitrogen), PVDF membranes (Bio-Rad) were blocked with 5% BSA in Tris-buffered saline containing 0.1% Tween 20 (TBS-T) for one hour and incubated overnight at 4°C with primary antibodies (β-actin, 4967L, Cell Signaling; NFKB, ab16502, abcam) diluted at 1:1000 in 5% BSA. Membranes were washed with TBS-T then incubated with HRP-conjugated donkey anti-rabbit secondary antibody (NA934V, Cytiva) for 1 hour at room temperature. Membranes were then washed again with TBS-T and treated with Super Signal West Pico chemiluminescent substrate (34580, Thermo Fisher). Membranes were imaged on iBright 1500.

Viability Staining

Viability of HK-2 cells was validated by Trypan Blue staining.

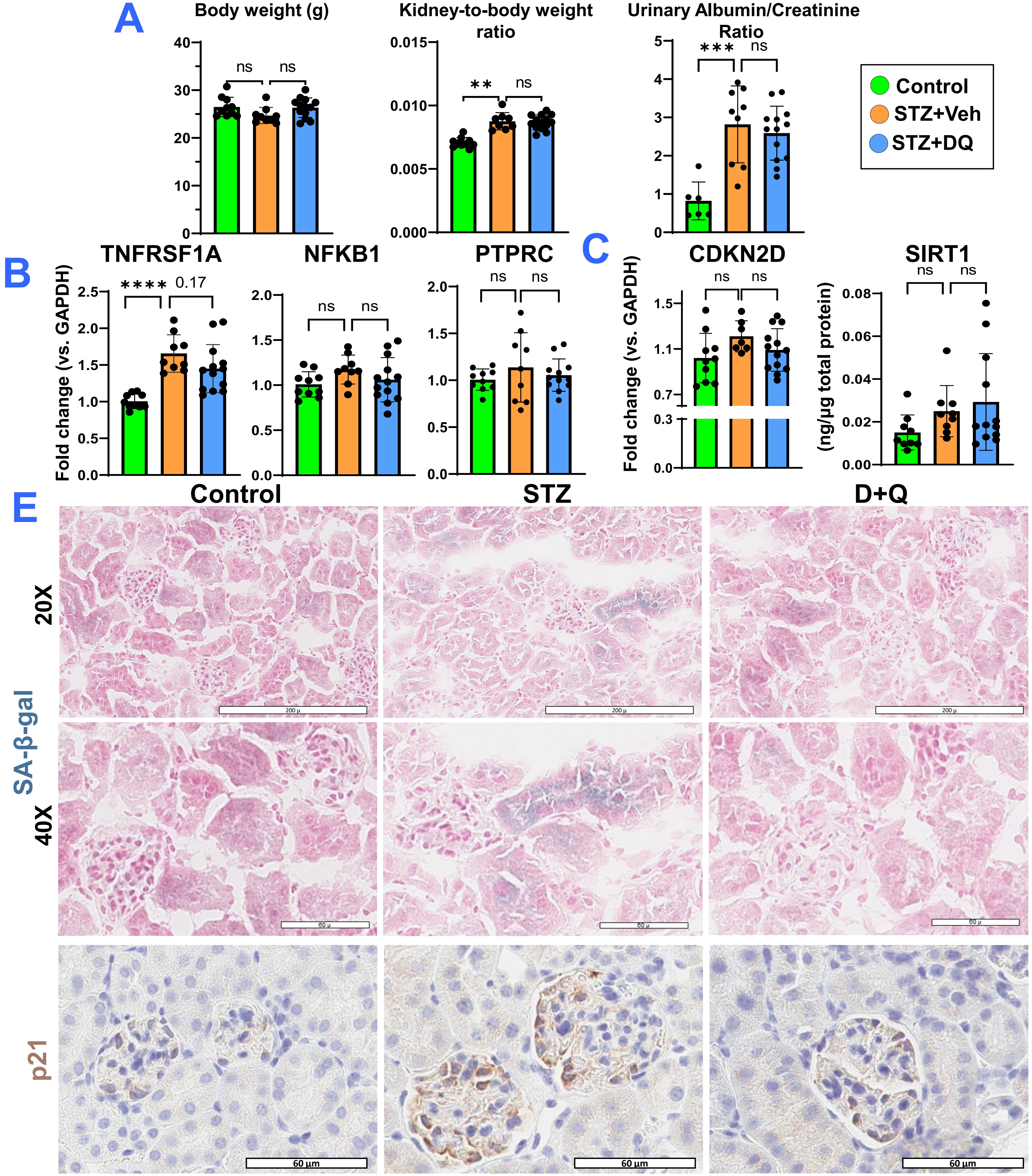

Supplemental Figure S1

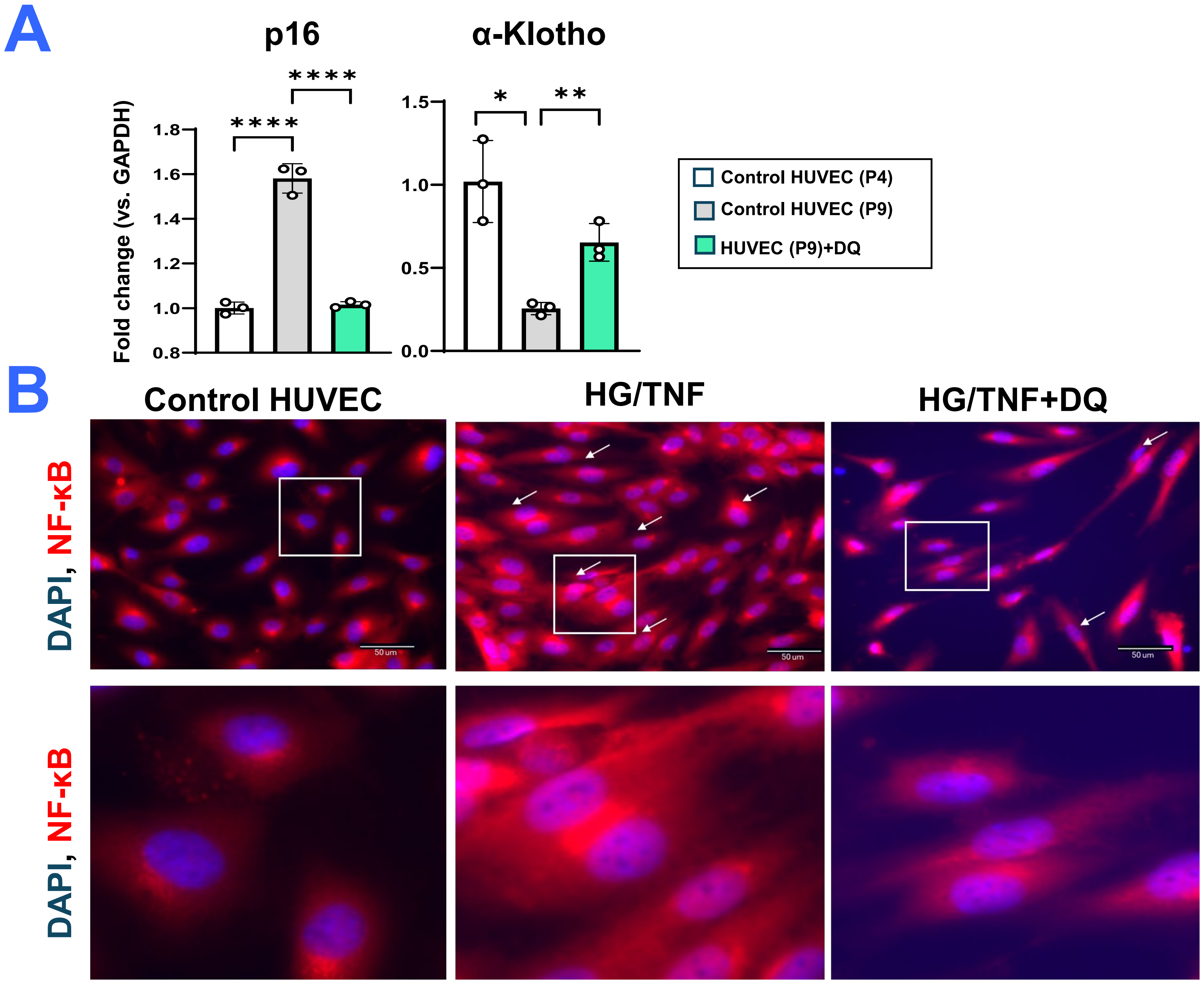

Supplemental Figure S2

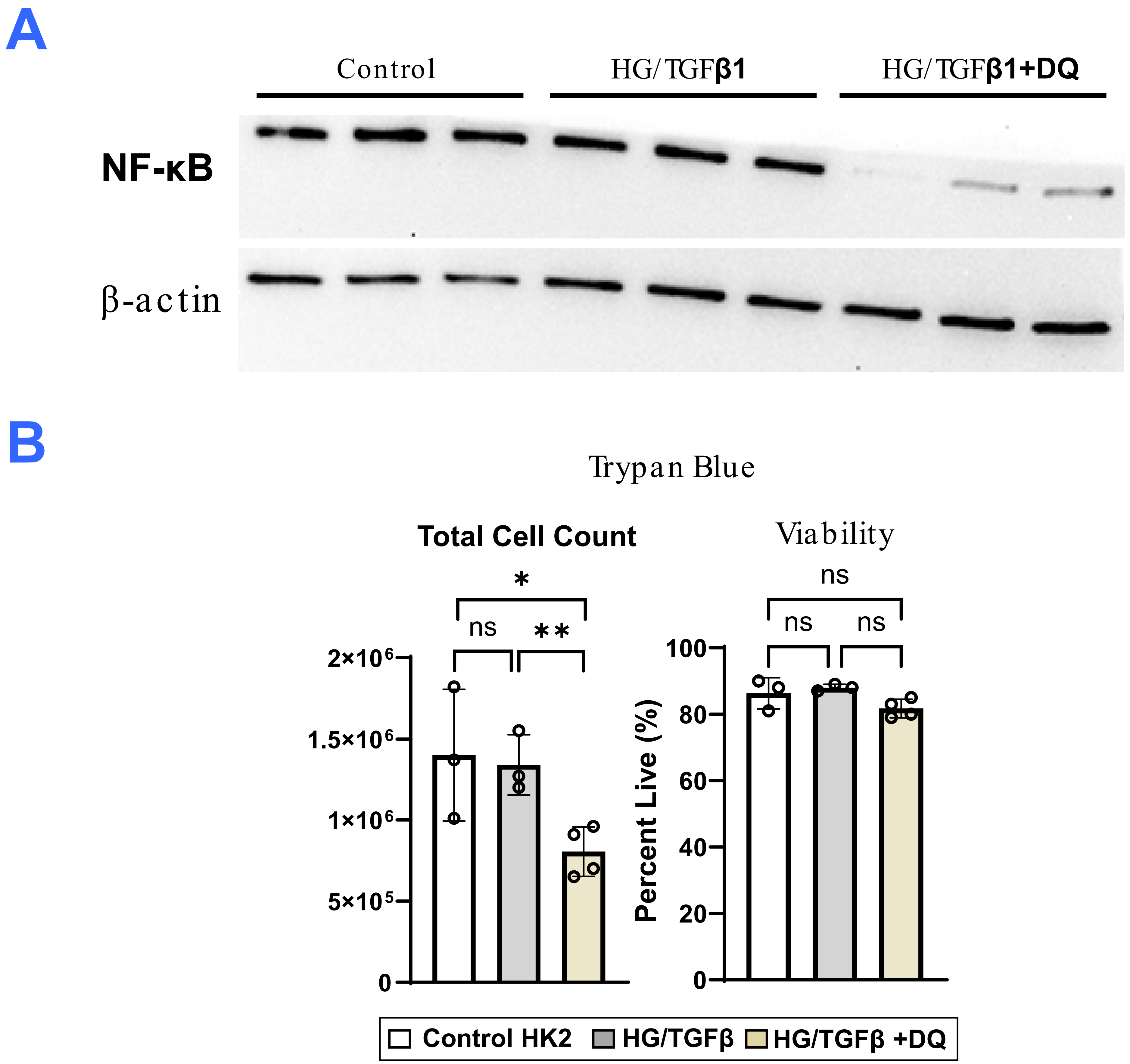

Supplemental Figure S3
